# (Dodecafluorosubphthalocyaninato)(4-methylphenolato)boron(III)

**DOI:** 10.1107/S1600536810042716

**Published:** 2010-11-06

**Authors:** Andrew S. Paton, Graham E. Morse, Jozef F. Maka, Alan J. Lough, Timothy P. Bender

**Affiliations:** a Department of Chemical Engineering & Applied Chemistry, University of Toronto, 200 College Street, Rm. 225, Toronto, Ontario, Canada M5S 3E5; b Department of Chemistry, University of Toronto, 80 St George St, Toronto, Ontario, Canada M5S 3H6

## Abstract

In the title compound, C_31_H_7_BF_12_N_6_O, mol­ecules are arranged into one-dimensional columns with an inter­molecular B⋯B distance of 5.3176 (8) Å. Bowl-shaped mol­ecules are arranged within the columns in a concave bowl-to-ligand arrangement separated by a ring centroid distance of 3.532 (2) Å between the benzene ring of the 4-methyl­phen­oxy ligand and one of the three five-membered rings of a symmetry-related mol­ecule.

## Related literature

For a general review of boronsubphthalocyanine compounds (BsubPcs), see: Claessens *et al.* (2002 ▶). For the application of BsubPcs in organic light-emitting diodes, see: Morse *et al.* (2010*a* ▶) and references cited therein. For applications of BsubPcs in organic solar cells, see: Gommans *et al.* (2009 ▶). For the first reported synthesis, characterization and crystal structure of PhO-F_12_BsubPc, see: Claessens & Torres (2002 ▶). For a synthetic process to obtain the precursor compound, Br-F_12_BsubPc, see: Sharman & van Lier (2005 ▶); Morse *et al.* (2010*b* ▶).
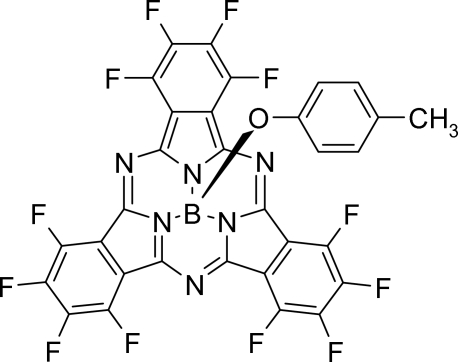


## Experimental

### 

#### Crystal data

C_31_H_7_BF_12_N_6_O
*M* *_r_* = 718.24
Monoclinic, 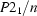

*a* = 14.6522 (5) Å
*b* = 10.5510 (6) Å
*c* = 18.0010 (7) Å
β = 96.663 (3)°
*V* = 2764.1 (2) Å^3^
*Z* = 4
Mo *K*α radiation
μ = 0.16 mm^−1^
*T* = 150 K
0.46 × 0.42 × 0.34 mm

#### Data collection

Nonius KappaCCD diffractometer
Absorption correction: multi-scan (*SORTAV*; Blessing, 1995 ▶) *T*_min_ = 0.764, *T*_max_ = 0.959
16887 measured reflections
6256 independent reflections
3481 reflections with *I* > 2σ(*I*)
*R*_int_ = 0.053

#### Refinement

*R*[*F* ^2^ > 2σ(*F* ^2^)] = 0.057
*wR*(*F* ^2^) = 0.172
*S* = 1.03
6256 reflections
460 parameters
H-atom parameters constrained
Δρ_max_ = 0.30 e Å^−3^
Δρ_min_ = −0.30 e Å^−3^

### 

Data collection: *COLLECT* (Nonius, 2002 ▶); cell refinement: *DENZO-SMN* (Otwinowski & Minor, 1997 ▶); data reduction: *DENZO-SMN*; program(s) used to solve structure: *SIR92* (Altomare *et al.*, 1994 ▶); program(s) used to refine structure: *SHELXTL* (Sheldrick, 2008 ▶); molecular graphics: *PLATON* (Spek, 2009 ▶) and *Mercury* (Macrae *et al.*, 2008 ▶); software used to prepare material for publication: *SHELXTL*.

## Supplementary Material

Crystal structure: contains datablocks I, global. DOI: 10.1107/S1600536810042716/jj2067sup1.cif

Structure factors: contains datablocks I. DOI: 10.1107/S1600536810042716/jj2067Isup2.hkl

Additional supplementary materials:  crystallographic information; 3D view; checkCIF report

## Figures and Tables

**Fig. 1.**
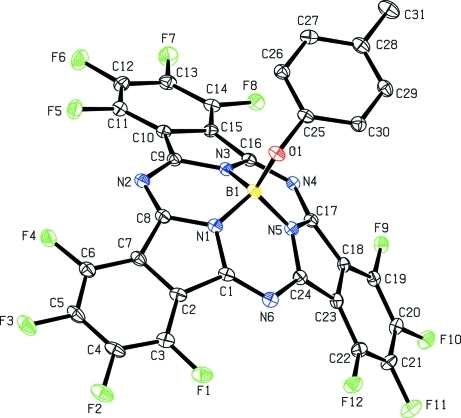
The molecular structure of the title compound with displacement ellipsoids drawn at the 30% probability level.

**Fig. 2.**
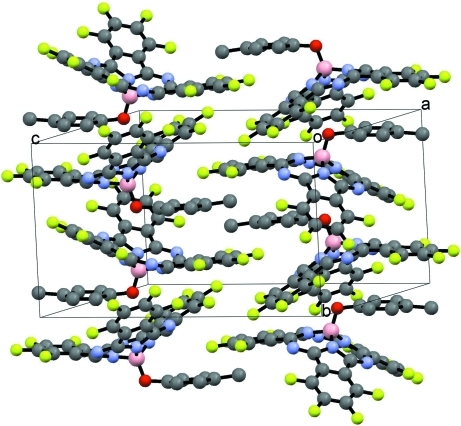
Part of the crystal structure of the title compound.

**Fig. 3.**
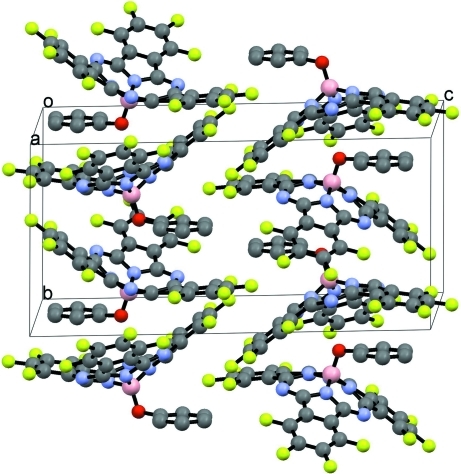
Part of the crystal structure of PhO-F12BsubPc (Morse et al, 2010a).

## supplementary crystallographic information

### Comment

Boronsubphthalocyanine (BsubPc) is unique amongst all phthalocyanines (Pcs) as only boron templates the formation of its cone-shaped macrocyclic aromatic ligand (Claessens *et al.*, 2002). Recently, BsubPcs have been shown to be useful as functional solid state materials in organic solar cells (Gommans *et al.*, 2009) and organic light emitting diodes (Morse *et al.*, 2010*a*). Two subclasses of BsubPcs are commonly used – dodecahydrogenated and dodecafluorinated – and they are generally applied as either their halo or phenoxy derivatives. However, little is known about their arrangement in the solid state, which is of interest to those who want to engineer functional devices containing BsubPcs.

We have synthesized 4-methylphenoxydodecafluoroboronsubphthalocyanine (4-MePhO-F_12_BsubPc) and obtained single crystals using a solvent diffusion method. The molecular structure of the title compound is shown in Fig. 1. In the crystal structure molecules are arranged into one-dimensional columns aligned approximately with the B—O bonds. (Fig. 2 b) with an intermolecular B···B distance of 5.3176 (8) Å (-*x* + 3/2, *y* + 1/2, -*z* + 1/2). Bowl-shaped molecules are arranged within the columns in a concave bowl to ligand arrangement separated by a ring centroid distance of 3.532 (2)Å between the benzene ring of the 4-methylphenoxy ligand (C25—C30) and one of the three five membered rings (N1/C1/C2/C7/C8) of a molecule at 3/2 - *x*,1/2 + *y*,1/2 - *z* (see Fig. 2).

A closely related compound, phenoxydodecafluoroboronsubphthalocyanine (PhO-F_12_BsubPc) has been previously synthesized (Claessens & Torres *et al.*, 2002; Morse *et al.* 2010*a*) and its crystal structures reported. In each case, with crystals grown under different conditions. As in the title compound, in the crystal structure of PhO-F_12_BsubPc, molecules arrange into one-dimensional columns again approximately aligned with the B—O bond regardless of the method of growth. The crystal structure of PhO-F_12_BsubPc (Morse *et al.*, 2010*a*) is re-illustrated for reference (Fig. 3). The intermolecular B···B distance in PhO-F_12_BsubPc is 5.3379 (7) Å (-*x* + 2, *y* + 1/2, -*z* + 3/2). a

The crystal structure of the title compound in addition to those of PhO-F_12_BsubPc suggest the arrangement of phenoxy-F_12_BsubPcs in the solid state may be predominant. In an effort to confirm or refute this, and to test the dependence on the nature of the alkyl substituent on the phenoxy group, we attempted to grow single crystals of 4 - *t*-butylphenoxydodecafluoroboronsubphthalocyanine. Unfortunately we found this derivative very soluble in organic solvents and were not able obtain single crystals as of yet.

### Experimental

Br-F_12_BsubPc was synthesized according to Morse *et al.* (2010*a*) which is an adaptation of the method of Sharman *et al.* (2005). For its crystal structure see Morse *et al.* (2010*b*).

4-MePhO-F_12_BsubPc. A solution of 1.00 g of (Br-F_12_BsubPc) in 5 ml of toluene was mixed with 0.78 g of 4-methylphenol. The mixture was stirred and heated to reflux under argon for 8 h. Reaction was determined complete *via* HPLC (RP-18 column, acetonitrile mobile phase 1.2 ml/min). The crude product was purified first by dissolving the product in toluene (300 ml) and extracting with 3.0 *M* KOH solution in distilled water (3 *x* 300 ml). The solvent was evaporated under vacuum and the product purified on a Kauffman column of alumina (absorbent) and dichloromethane (eluent). The Kauffman column was run overnight and subsequently the dichloromethane was removed under reduced pressure leaving a dark pink powder (0.52 g, 0.00072 mol, 44% yield). Crystals of the title compound were grown by slow diffusion of heptane into a solution of the title compound in benzene.

### Crystal data

**Table:**

C_31_H_7_BF_12_N_6_O	*F*(000) = 1424
*M**_r_* = 718.24	*D*_x_ = 1.726 Mg m^−^^3^
Monoclinic, *P*2_1_/*n*	Mo *K*α radiation, λ = 0.71073 Å
Hall symbol: -P 2yn	Cell parameters from 16887 reflections
*a* = 14.6522 (5) Å	θ = 2.6–27.5°
*b* = 10.5510 (6) Å	µ = 0.16 mm^−^^1^
*c* = 18.0010 (7) Å	*T* = 150 K
β = 96.663 (3)°	Block, purple
*V* = 2764.1 (2) Å^3^	0.46 × 0.42 × 0.34 mm
*Z* = 4	

### Data collection

**Table:**

Nonius KappaCCD diffractometer	6256 independent reflections
Radiation source: fine-focus sealed tube	3481 reflections with *I* > 2σ(*I*)
graphite	*R*_int_ = 0.053
Detector resolution: 9 pixels mm^-1^	θ_max_ = 27.5°, θ_min_ = 2.6°
φ scans and ω scans with κ offsets	*h* = −18→18
Absorption correction: multi-scan (*SORTAV*; Blessing, 1995)	*k* = −13→13
*T*_min_ = 0.764, *T*_max_ = 0.959	*l* = −23→23
16887 measured reflections	

### Refinement

**Table:**

Refinement on *F*^2^	Primary atom site location: structure-invariant direct methods
Least-squares matrix: full	Secondary atom site location: difference Fourier map
*R*[*F*^2^ > 2σ(*F*^2^)] = 0.057	Hydrogen site location: inferred from neighbouring sites
*wR*(*F*^2^) = 0.172	H-atom parameters constrained
*S* = 1.03	*w* = 1/[σ^2^(*F*_o_^2^) + (0.0902*P*)^2^ + 0.2514*P*] where *P* = (*F*_o_^2^ + 2*F*_c_^2^)/3
6256 reflections	(Δ/σ)_max_ = 0.001
460 parameters	Δρ_max_ = 0.30 e Å^−^^3^
0 restraints	Δρ_min_ = −0.30 e Å^−^^3^

### Special details

**Table:**

Geometry. All e.s.d.'s (except the e.s.d. in the dihedral angle between two l.s. planes) are estimated using the full covariance matrix. The cell e.s.d.'s are taken into account individually in the estimation of e.s.d.'s in distances, angles and torsion angles; correlations between e.s.d.'s in cell parameters are only used when they are defined by crystal symmetry. An approximate (isotropic) treatment of cell e.s.d.'s is used for estimating e.s.d.'s involving l.s. planes.
Refinement. Refinement of *F*^2^ against ALL reflections. The weighted *R*-factor *wR* and goodness of fit *S* are based on *F*^2^, conventional *R*-factors *R* are based on *F*, with *F* set to zero for negative *F*^2^. The threshold expression of *F*^2^ > σ(*F*^2^) is used only for calculating *R*-factors(gt) *etc*. and is not relevant to the choice of reflections for refinement. *R*-factors based on *F*^2^ are statistically about twice as large as those based on *F*, and *R*- factors based on ALL data will be even larger.

### Fractional atomic coordinates and isotropic or equivalent isotropic displacement parameters (Å^2^)

**Table:**

	*x*	*y*	*z*	*U*_iso_*/*U*_eq_	
F1	0.53576 (13)	0.27010 (17)	0.50181 (10)	0.0538 (5)	
F2	0.62959 (14)	0.29013 (18)	0.63991 (10)	0.0619 (5)	
F3	0.81470 (14)	0.29484 (19)	0.65863 (9)	0.0643 (6)	
F4	0.91201 (12)	0.28163 (17)	0.54072 (9)	0.0516 (5)	
F5	1.08623 (11)	0.37830 (19)	0.33494 (9)	0.0536 (5)	
F6	1.17681 (11)	0.5008 (2)	0.23366 (10)	0.0627 (6)	
F7	1.09226 (12)	0.56366 (19)	0.09845 (10)	0.0577 (5)	
F8	0.91311 (11)	0.50438 (15)	0.05849 (8)	0.0425 (4)	
F9	0.57526 (10)	0.56024 (14)	0.03931 (8)	0.0364 (4)	
F10	0.40881 (11)	0.66504 (17)	0.05162 (9)	0.0476 (4)	
F11	0.31482 (11)	0.60452 (19)	0.16583 (11)	0.0588 (5)	
F12	0.38054 (11)	0.43226 (18)	0.27000 (9)	0.0506 (5)	
O1	0.72879 (13)	0.09082 (16)	0.22748 (10)	0.0338 (5)	
N1	0.72624 (15)	0.2315 (2)	0.33623 (12)	0.0306 (5)	
N2	0.88549 (16)	0.2812 (2)	0.36128 (12)	0.0346 (6)	
N3	0.80863 (14)	0.2979 (2)	0.23845 (11)	0.0287 (5)	
N4	0.73305 (14)	0.4059 (2)	0.13397 (11)	0.0297 (5)	
N5	0.64793 (14)	0.2999 (2)	0.22105 (11)	0.0294 (5)	
N6	0.56821 (15)	0.2881 (2)	0.32889 (12)	0.0348 (6)	
C1	0.64781 (19)	0.2520 (2)	0.36801 (15)	0.0319 (6)	
C2	0.67586 (19)	0.2593 (3)	0.44816 (15)	0.0344 (7)	
C3	0.6281 (2)	0.2706 (3)	0.50962 (16)	0.0407 (7)	
C4	0.6750 (2)	0.2810 (3)	0.57947 (16)	0.0470 (8)	
C5	0.7710 (3)	0.2829 (3)	0.58906 (15)	0.0469 (8)	
C6	0.8201 (2)	0.2749 (3)	0.52897 (16)	0.0410 (7)	
C7	0.7738 (2)	0.2598 (3)	0.45805 (15)	0.0353 (7)	
C8	0.80436 (19)	0.2517 (3)	0.38416 (14)	0.0323 (6)	
C9	0.88473 (18)	0.3097 (3)	0.28843 (14)	0.0319 (6)	
C10	0.95087 (18)	0.3763 (3)	0.24878 (15)	0.0341 (6)	
C11	1.04161 (19)	0.4099 (3)	0.26770 (16)	0.0399 (7)	
C12	1.08789 (19)	0.4732 (3)	0.21697 (17)	0.0435 (7)	
C13	1.0438 (2)	0.5054 (3)	0.14710 (16)	0.0414 (7)	
C14	0.95285 (19)	0.4748 (3)	0.12730 (15)	0.0350 (6)	
C15	0.90513 (17)	0.4115 (2)	0.17733 (14)	0.0299 (6)	
C16	0.81024 (17)	0.3691 (2)	0.17553 (14)	0.0298 (6)	
C17	0.65355 (17)	0.3781 (2)	0.16128 (14)	0.0303 (6)	
C18	0.56452 (17)	0.4394 (2)	0.14843 (14)	0.0298 (6)	
C19	0.52869 (18)	0.5275 (3)	0.09645 (14)	0.0317 (6)	
C20	0.44506 (18)	0.5812 (3)	0.10268 (16)	0.0363 (7)	
C21	0.39572 (18)	0.5493 (3)	0.16124 (17)	0.0405 (7)	
C22	0.42956 (18)	0.4611 (3)	0.21442 (16)	0.0386 (7)	
C23	0.51408 (18)	0.4049 (2)	0.20818 (15)	0.0323 (6)	
C24	0.57225 (18)	0.3200 (3)	0.25680 (14)	0.0319 (6)	
C25	0.74723 (19)	0.0818 (2)	0.15342 (15)	0.0330 (6)	
C26	0.8368 (2)	0.0748 (3)	0.13729 (16)	0.0368 (7)	
H26A	0.8855	0.0669	0.1767	0.044*	
C27	0.8561 (2)	0.0793 (3)	0.06386 (16)	0.0404 (7)	
H27A	0.9181	0.0752	0.0535	0.048*	
C28	0.7860 (2)	0.0899 (3)	0.00501 (16)	0.0421 (7)	
C29	0.6966 (2)	0.0895 (3)	0.02170 (16)	0.0396 (7)	
H29A	0.6478	0.0917	−0.0180	0.048*	
C30	0.6757 (2)	0.0858 (3)	0.09520 (16)	0.0366 (7)	
H30A	0.6136	0.0860	0.1055	0.044*	
C31	0.8089 (3)	0.1065 (3)	−0.07462 (17)	0.0565 (9)	
H31A	0.7518	0.1119	−0.1088	0.085*	
H31B	0.8450	0.0337	−0.0884	0.085*	
H31C	0.8445	0.1844	−0.0780	0.085*	
B1	0.7282 (2)	0.2205 (3)	0.25372 (17)	0.0310 (7)	

### Atomic displacement parameters (Å^2^)

**Table:**

	*U*^11^	*U*^22^	*U*^33^	*U*^12^	*U*^13^	*U*^23^
F1	0.0586 (12)	0.0578 (12)	0.0494 (11)	0.0048 (9)	0.0245 (9)	0.0038 (9)
F2	0.0932 (14)	0.0602 (13)	0.0372 (10)	0.0038 (11)	0.0279 (10)	0.0013 (9)
F3	0.0989 (15)	0.0645 (13)	0.0276 (9)	0.0151 (11)	−0.0004 (9)	−0.0035 (8)
F4	0.0577 (12)	0.0556 (12)	0.0390 (10)	0.0108 (9)	−0.0050 (8)	−0.0072 (8)
F5	0.0401 (10)	0.0752 (13)	0.0436 (10)	−0.0023 (9)	−0.0037 (8)	0.0029 (9)
F6	0.0362 (10)	0.0909 (16)	0.0612 (12)	−0.0162 (10)	0.0067 (9)	−0.0018 (11)
F7	0.0476 (10)	0.0745 (14)	0.0540 (11)	−0.0162 (9)	0.0186 (9)	0.0036 (10)
F8	0.0486 (10)	0.0449 (10)	0.0351 (9)	−0.0025 (8)	0.0099 (7)	0.0056 (8)
F9	0.0389 (9)	0.0381 (9)	0.0318 (8)	−0.0049 (7)	0.0019 (7)	0.0055 (7)
F10	0.0438 (9)	0.0486 (11)	0.0490 (10)	0.0067 (8)	0.0001 (8)	0.0148 (9)
F11	0.0384 (10)	0.0699 (13)	0.0693 (13)	0.0131 (9)	0.0124 (9)	0.0191 (10)
F12	0.0383 (9)	0.0670 (12)	0.0489 (10)	0.0020 (8)	0.0157 (8)	0.0106 (9)
O1	0.0487 (12)	0.0254 (10)	0.0288 (10)	−0.0013 (8)	0.0106 (9)	0.0009 (8)
N1	0.0390 (13)	0.0257 (12)	0.0278 (12)	0.0014 (10)	0.0063 (10)	0.0030 (9)
N2	0.0429 (14)	0.0325 (13)	0.0285 (12)	0.0076 (11)	0.0042 (10)	0.0002 (10)
N3	0.0342 (12)	0.0260 (12)	0.0259 (11)	0.0017 (10)	0.0034 (9)	−0.0004 (9)
N4	0.0328 (12)	0.0297 (12)	0.0268 (11)	−0.0015 (10)	0.0035 (10)	−0.0022 (10)
N5	0.0345 (12)	0.0274 (12)	0.0264 (11)	−0.0019 (10)	0.0048 (10)	0.0025 (10)
N6	0.0394 (13)	0.0303 (13)	0.0358 (13)	−0.0041 (11)	0.0093 (11)	0.0037 (10)
C1	0.0423 (16)	0.0252 (14)	0.0301 (14)	0.0004 (12)	0.0120 (13)	0.0027 (11)
C2	0.0470 (17)	0.0252 (14)	0.0327 (15)	0.0050 (12)	0.0123 (13)	0.0046 (12)
C3	0.0513 (19)	0.0336 (16)	0.0393 (17)	0.0032 (14)	0.0137 (15)	0.0049 (13)
C4	0.077 (2)	0.0359 (17)	0.0316 (16)	0.0048 (16)	0.0229 (16)	0.0015 (13)
C5	0.079 (2)	0.0369 (17)	0.0247 (15)	0.0089 (16)	0.0047 (15)	−0.0004 (13)
C6	0.055 (2)	0.0323 (16)	0.0348 (16)	0.0096 (14)	0.0015 (14)	0.0001 (13)
C7	0.0508 (18)	0.0271 (14)	0.0278 (15)	0.0045 (13)	0.0046 (13)	−0.0001 (12)
C8	0.0420 (17)	0.0262 (14)	0.0287 (15)	0.0048 (12)	0.0048 (13)	0.0022 (11)
C9	0.0361 (15)	0.0295 (15)	0.0300 (14)	0.0075 (12)	0.0031 (12)	−0.0003 (12)
C10	0.0351 (15)	0.0313 (15)	0.0364 (15)	0.0041 (12)	0.0058 (12)	−0.0057 (13)
C11	0.0390 (17)	0.0438 (18)	0.0368 (16)	0.0030 (14)	0.0039 (14)	−0.0047 (14)
C12	0.0295 (15)	0.0519 (19)	0.0499 (18)	−0.0036 (14)	0.0087 (14)	−0.0064 (16)
C13	0.0413 (17)	0.0409 (18)	0.0449 (17)	−0.0050 (14)	0.0177 (14)	−0.0026 (14)
C14	0.0415 (16)	0.0318 (15)	0.0334 (15)	0.0030 (13)	0.0119 (13)	−0.0027 (13)
C15	0.0326 (14)	0.0270 (14)	0.0308 (14)	0.0035 (11)	0.0070 (12)	0.0020 (11)
C16	0.0361 (15)	0.0259 (14)	0.0283 (13)	0.0015 (12)	0.0068 (12)	0.0000 (12)
C17	0.0343 (15)	0.0289 (15)	0.0279 (14)	0.0005 (12)	0.0048 (11)	0.0002 (12)
C18	0.0321 (14)	0.0274 (14)	0.0289 (14)	−0.0048 (12)	−0.0003 (12)	−0.0009 (11)
C19	0.0322 (15)	0.0343 (15)	0.0286 (14)	−0.0090 (12)	0.0044 (12)	0.0007 (12)
C20	0.0315 (15)	0.0361 (16)	0.0399 (16)	−0.0036 (12)	−0.0024 (13)	0.0054 (13)
C21	0.0266 (15)	0.0473 (18)	0.0480 (18)	0.0026 (13)	0.0060 (13)	0.0060 (15)
C22	0.0314 (15)	0.0451 (17)	0.0409 (16)	−0.0057 (13)	0.0107 (13)	0.0018 (14)
C23	0.0322 (14)	0.0318 (15)	0.0321 (15)	−0.0040 (12)	0.0002 (12)	−0.0006 (12)
C24	0.0326 (14)	0.0311 (15)	0.0323 (14)	−0.0055 (12)	0.0055 (12)	0.0000 (12)
C25	0.0468 (17)	0.0227 (14)	0.0309 (15)	0.0022 (12)	0.0100 (13)	0.0010 (11)
C26	0.0440 (17)	0.0296 (15)	0.0369 (16)	0.0037 (13)	0.0046 (13)	−0.0011 (12)
C27	0.0435 (17)	0.0340 (16)	0.0460 (18)	0.0012 (13)	0.0150 (15)	−0.0014 (14)
C28	0.060 (2)	0.0288 (16)	0.0379 (17)	−0.0007 (14)	0.0098 (15)	−0.0015 (13)
C29	0.0547 (19)	0.0323 (16)	0.0309 (15)	0.0002 (14)	0.0007 (14)	0.0005 (12)
C30	0.0402 (16)	0.0286 (15)	0.0412 (17)	−0.0039 (12)	0.0054 (13)	−0.0037 (12)
C31	0.085 (3)	0.049 (2)	0.0384 (18)	−0.0006 (18)	0.0210 (17)	0.0022 (15)
B1	0.0375 (18)	0.0295 (17)	0.0263 (15)	0.0006 (14)	0.0045 (14)	0.0015 (13)

### Geometric parameters (Å, °)

**Table:**

F1—C3	1.343 (3)	C5—C6	1.370 (4)
F2—C4	1.343 (3)	C6—C7	1.383 (4)
F3—C5	1.345 (3)	C7—C8	1.454 (4)
F4—C6	1.341 (3)	C9—C10	1.450 (4)
F5—C11	1.349 (3)	C10—C11	1.380 (4)
F6—C12	1.335 (3)	C10—C15	1.429 (4)
F7—C13	1.339 (3)	C11—C12	1.373 (4)
F8—C14	1.343 (3)	C12—C13	1.388 (4)
F9—C19	1.344 (3)	C13—C14	1.377 (4)
F10—C20	1.340 (3)	C14—C15	1.375 (4)
F11—C21	1.332 (3)	C15—C16	1.457 (4)
F12—C22	1.333 (3)	C17—C18	1.450 (4)
O1—C25	1.394 (3)	C18—C19	1.379 (4)
O1—B1	1.448 (4)	C18—C23	1.421 (4)
N1—C1	1.359 (3)	C19—C20	1.367 (4)
N1—C8	1.368 (3)	C20—C21	1.387 (4)
N1—B1	1.493 (4)	C21—C22	1.385 (4)
N2—C8	1.339 (4)	C22—C23	1.389 (4)
N2—C9	1.344 (3)	C23—C24	1.457 (4)
N3—C9	1.355 (3)	C25—C26	1.379 (4)
N3—C16	1.361 (3)	C25—C30	1.394 (4)
N3—B1	1.485 (4)	C26—C27	1.384 (4)
N4—C16	1.339 (3)	C26—H26A	0.9500
N4—C17	1.348 (3)	C27—C28	1.392 (4)
N5—C24	1.362 (3)	C27—H27A	0.9500
N5—C17	1.366 (3)	C28—C29	1.378 (4)
N5—B1	1.507 (4)	C28—C31	1.519 (4)
N6—C1	1.345 (3)	C29—C30	1.393 (4)
N6—C24	1.349 (3)	C29—H29A	0.9500
C1—C2	1.456 (4)	C30—H30A	0.9500
C2—C3	1.381 (4)	C31—H31A	0.9800
C2—C7	1.426 (4)	C31—H31B	0.9800
C3—C4	1.366 (4)	C31—H31C	0.9800
C4—C5	1.397 (5)		
			
C25—O1—B1	112.7 (2)	N4—C16—N3	122.0 (2)
C1—N1—C8	113.3 (2)	N4—C16—C15	131.7 (2)
C1—N1—B1	123.1 (2)	N3—C16—C15	105.0 (2)
C8—N1—B1	121.9 (2)	N4—C17—N5	123.5 (2)
C8—N2—C9	116.4 (2)	N4—C17—C18	130.1 (2)
C9—N3—C16	114.2 (2)	N5—C17—C18	105.0 (2)
C9—N3—B1	122.5 (2)	C19—C18—C23	119.7 (2)
C16—N3—B1	123.2 (2)	C19—C18—C17	132.3 (2)
C16—N4—C17	116.3 (2)	C23—C18—C17	107.6 (2)
C24—N5—C17	113.9 (2)	F9—C19—C20	119.7 (2)
C24—N5—B1	122.9 (2)	F9—C19—C18	120.4 (2)
C17—N5—B1	121.8 (2)	C20—C19—C18	119.9 (2)
C1—N6—C24	116.2 (2)	F10—C20—C19	120.4 (3)
N6—C1—N1	123.3 (2)	F10—C20—C21	118.8 (2)
N6—C1—C2	129.2 (2)	C19—C20—C21	120.9 (3)
N1—C1—C2	105.7 (2)	F11—C21—C22	120.1 (3)
C3—C2—C7	119.8 (3)	F11—C21—C20	119.2 (3)
C3—C2—C1	133.4 (3)	C22—C21—C20	120.8 (3)
C7—C2—C1	106.8 (2)	F12—C22—C21	119.4 (3)
F1—C3—C4	119.3 (3)	F12—C22—C23	121.8 (3)
F1—C3—C2	120.9 (3)	C21—C22—C23	118.8 (3)
C4—C3—C2	119.8 (3)	C22—C23—C18	119.9 (2)
F2—C4—C3	120.5 (3)	C22—C23—C24	132.7 (3)
F2—C4—C5	119.0 (3)	C18—C23—C24	107.0 (2)
C3—C4—C5	120.5 (3)	N6—C24—N5	123.0 (2)
F3—C5—C6	120.3 (3)	N6—C24—C23	130.1 (2)
F3—C5—C4	118.7 (3)	N5—C24—C23	105.3 (2)
C6—C5—C4	121.0 (3)	C26—C25—O1	120.0 (2)
F4—C6—C5	118.8 (3)	C26—C25—C30	119.6 (3)
F4—C6—C7	121.8 (3)	O1—C25—C30	120.3 (2)
C5—C6—C7	119.4 (3)	C25—C26—C27	120.2 (3)
C6—C7—C2	119.6 (3)	C25—C26—H26A	119.9
C6—C7—C8	133.0 (3)	C27—C26—H26A	119.9
C2—C7—C8	107.3 (2)	C26—C27—C28	121.0 (3)
N2—C8—N1	123.4 (2)	C26—C27—H27A	119.5
N2—C8—C7	130.2 (3)	C28—C27—H27A	119.5
N1—C8—C7	105.2 (2)	C29—C28—C27	118.0 (3)
N2—C9—N3	122.7 (2)	C29—C28—C31	121.7 (3)
N2—C9—C10	131.2 (2)	C27—C28—C31	120.2 (3)
N3—C9—C10	105.3 (2)	C28—C29—C30	121.8 (3)
C11—C10—C15	119.6 (3)	C28—C29—H29A	119.1
C11—C10—C9	133.0 (3)	C30—C29—H29A	119.1
C15—C10—C9	107.4 (2)	C29—C30—C25	119.1 (3)
F5—C11—C12	119.5 (3)	C29—C30—H30A	120.4
F5—C11—C10	120.7 (3)	C25—C30—H30A	120.4
C12—C11—C10	119.8 (3)	C28—C31—H31A	109.5
F6—C12—C11	120.1 (3)	C28—C31—H31B	109.5
F6—C12—C13	119.4 (3)	H31A—C31—H31B	109.5
C11—C12—C13	120.5 (3)	C28—C31—H31C	109.5
F7—C13—C14	120.6 (3)	H31A—C31—H31C	109.5
F7—C13—C12	118.6 (3)	H31B—C31—H31C	109.5
C14—C13—C12	120.8 (3)	O1—B1—N3	115.1 (2)
F8—C14—C15	121.2 (2)	O1—B1—N1	113.5 (2)
F8—C14—C13	119.1 (2)	N3—B1—N1	104.4 (2)
C15—C14—C13	119.6 (3)	O1—B1—N5	115.6 (2)
C14—C15—C10	119.6 (2)	N3—B1—N5	102.8 (2)
C14—C15—C16	133.7 (2)	N1—B1—N5	103.9 (2)
C10—C15—C16	106.7 (2)		
